# Bohr-type inequalities of analytic functions

**DOI:** 10.1186/s13660-018-1937-y

**Published:** 2018-12-13

**Authors:** Ming-Sheng Liu, Yin-Miao Shang, Jun-Feng Xu

**Affiliations:** 1 School of Mathematical Sciences, South China Normal University, Guangzhou, China; 2 Department of Mathematics, Wuyi University, Jiangmen, China

**Keywords:** 30C45, 30A10, 30H05, Bounded analytic function, Bohr radius, Bohr–Rogosinski sum, Bohr-type radius

## Abstract

In this paper, we investigate the Bohr-type radii for several different forms of Bohr-type inequalities of analytic functions in the unit disk, we also investigate the Bohr-type radius of the alternating series associated with the Taylor series of analytic functions. We will prove that most of the results are sharp.

## Introduction and preliminaries

Bohr’s inequality states that if

1.1 $$ f(z)=\sum_{k=0}^{\infty}a_{k}z^{k} $$

is analytic in the unit disk $D=\{z\in\mathbb{C}||z|<1\}$ and $|f(z)|<1$ for all $z\in D$, then

1.2 $$ \sum_{k=0}^{\infty} \vert a_{k} \vert \vert z \vert ^{k}\leq1 $$

for all $|z|\leq\frac{1}{3}$. This inequality was discovered by Bohr in 1914 [6]. Bohr actually obtained the inequality for $|z|\leq \frac{1}{6}$, but subsequently later, Wiener, Riesz and Schur, independently established the inequality for $|z|\leq\frac{1}{3}$ and the constant 1/3 cannot be improved [12, 16, 17]. Other proofs were also given in [13, 14]. The problem was considered by Bohr when he was working on the absolute convergence problem for Dirichlet series of the form $\sum a_{n}n^{-s}$, but now it has become a very interesting problem. Bohr’s idea naturally extends to functions of several complex variables [1, 2, 5, 11] and a variety of results on Bohr’s theorem in higher dimensions appeared recently.

The majorant series $M_{f}(z)=\sum_{k=0}^{\infty}|a_{k}||z|^{k}$ belongs to a very important class of series of non-negative terms. In analogy to the Bohr radius, there is also the notion of the Rogosinski radius [10, 15], which is described as follows: If $f(z)=\sum_{k=0}^{\infty}a_{k}z^{k}$ is an analytic function in *D* such that $|f(z)|< 1$ in *D*, then, for every $N\geq1$, we have $|s_{N}(z)|<1$ in the disk $|z|<\frac{1}{2}$ and this radius is sharp, where $S_{N}(z)=\sum_{k=0}^{N-1}a_{k}z^{k}$ denotes the partial sums of *f*. There is a relevant quantity, which we call the Bohr–Rogosinski sum $R_{N}^{f}(z)$ of *f* defined by

1.3 $$ R_{N}^{f}(z):= \bigl\vert f(z) \bigr\vert +\sum _{k=N}^{\infty} \vert a_{k} \vert r^{k},\quad \vert z \vert =r. $$

We remark that, for $N=1$, this quantity is related to the classical Bohr sum in which $f(0)$ is replaced by $f(z)$. More recently, Kayumov and Ponnusamy [9] obtained the following result on the Bohr–Rogosinski radius for analytic functions.

### Theorem A

([9])

*Suppose that* $f(z)=\sum_{k=0}^{\infty }a_{k}z^{k}$ *is analytic in the unit disk* *D* *and* $|f(z)|< 1$ *in* *D*. *Then*

$$ \bigl\vert f(z) \bigr\vert +\sum_{k=N}^{\infty} \vert a_{k} \vert r^{k}\leq1\quad \textit{for } r\leq R_{N}, $$

*where* $R_{N}$ *is the positive root of the equation* $2(1+r)r^{N}-(1-r)^{2}=0$. *The radius* $R_{N}$ *is the best possible*. *Moreover*,

$$ \bigl\vert f(z) \bigr\vert ^{2}+\sum _{k=N}^{\infty} \vert a_{k} \vert r^{k}\leq1 \quad\textit{for } r\leq R'_{N}, $$

*where* $R'_{N}$ *is the positive root of the equation* $(1+r)r^{N}-(1-r)^{2}=0$. *The radius* $R'_{N}$ *is the best possible*.

In 2017, Ali, Barnard and Solynin defined the associated alternating series of series (1.1) as $A_{f}(z)=\sum_{k=0}^{\infty }(-1)^{k}|a_{k}||z|^{k}$, they obtained the following result in [4].

### Theorem B

([4])

*If* $|\sum_{k=0}^{\infty}a_{k}z^{k}|\leq 1$ *in* *D*, *then*

$$ \Biggl\vert \sum_{k=0}^{\infty}(-1)^{k} | a_{k}| \vert z \vert ^{k} \Biggr\vert \leq1 $$

*in the disk* $D_{1/\sqrt{3}}=\{z\in\mathbb{C}||z|<1/\sqrt{3}\}$. *The radius* $r=1/\sqrt{3}$ *is the best possible*.

### Theorem C

([3])

*If* $f(z)=\sum_{k=0}^{\infty }a_{nk}z^{nk}$ *is analytic in* *D* *satisfying* $\operatorname{ Re} f(z)\leq1$ *in* *D* *and* $f(0)=a_{0}$ *is positive*, *then* $M_{f}(r)\leq1$ *for* $0\leq r\leq 1/\sqrt[n]{3}$.

### Remark 1.1

By a simple calculation in Theorem A, we observe that $R_{1}=\sqrt{5}-2$ is unequal to $\frac{1}{3}$ when $|f(0)|$ is replaced by $|f(z)|$ in Bohr’s inequality. Therefore, it is interesting to note what will happen to the Bohr radius if we use higher order derivatives of $f(z)$ to replace some Taylor coefficients of analytic functions in Bohr’s inequality.

In this paper, we mainly study the Bohr-type radii for several forms of Bohr-type inequalities of analytic functions when the Taylor coefficients of classical Bohr inequality are partly replaced and when the Taylor coefficients of the classical Bohr inequality are completely replaced by the higher order derivatives of $f(z)$, respectively. We obtain the Bohr-type radii under certain conditions. Moreover, we also discuss the Bohr-type radius of the alternating series associated with the Taylor series of analytic functions.

In order to establish our main results, we need the following lemmas, which will play the key role in proving the main results of this paper.

### Lemma 1.2

([8])

*If* $\varphi(z)=\sum_{n=0}^{\infty }a_{n}z^{n}$ *is analytic and* $|\varphi(z)|\leq1$ *in the unit disk* *D*. *Then* $|a_{n}|\leq1-|a_{0}|^{2}$ *for all* $n=1,2,\ldots $ .

### Lemma 1.3

(Schwarz–Pick lemma)

*If* $\varphi(z)=\sum_{n=0}^{\infty }a_{n}z^{n}$ *is analytic and* $|\varphi(z)|< 1$ *in the unit disk* *D*. *Then*:

1. $\vert \varphi(z_{1})-\varphi(z_{2}) \vert / \vert 1-\overline{\varphi (z_{1})}\varphi(z_{2}) \vert \leq \vert z_{1}-z_{2} \vert / \vert 1-\overline {z_{1}}z_{2} \vert $ *holds for* $z_{1}, z_{2}\in D$, *and the equality holds for distinct* $z_{1}, z_{2}\in D$ *if and only if* *φ* *is a Möbius transformation*;
2. $|\varphi'(z)|\leq\frac{1-|\varphi(z)|^{2}}{1-|z|^{2}}$ *holds for* $z\in D$, *and the equality holds for some* $z\in U$ *if and only if* *f* *is a Möbius transformation*.

### Lemma 1.4

([7])

*If* $\varphi(z)=\sum_{n=0}^{\infty }a_{n}z^{n}$ *is analytic and* $|\varphi(z)|< 1$ *in* *D*. *Then*, *for all* $k=1,2,\ldots $ , *we have*

$$\bigl\vert \varphi^{(k)}(z) \bigr\vert \leq\frac{k!(1- \vert \varphi (z) \vert ^{2})}{(1- \vert z \vert ^{2})^{k}} \bigl(1+ \vert z \vert \bigr)^{k-1}, \quad \vert z \vert < 1. $$

### Lemma 1.5

([3])

*If* $p(z)=\sum_{k=0}^{\infty }p_{k}z^{k}$ *is analytic in* *D* *such that* $\operatorname{ Re} p(z)>0$ *in* *D*, *then* $|p_{k}|\leq2\operatorname{ Re} p_{0}$ *for all* $k\geq1$.

## Main results

We first provide a result involves computing Bohr-type radius for the analytic functions $f(z)$ for which $|a_{0}|$ and $|a_{1}|$ are replaced by $|f(z)|$ and $|f'(z)|$, respectively.

### Theorem 2.1

*Suppose that* $f(z)=\sum_{k=0}^{\infty}a_{k}z^{k}$ *is analytic in* *D* *and* $|f(z)|<1$ *in* *D*. *Then*

$$ \bigl\vert f(z) \bigr\vert + \bigl\vert f'(z) \bigr\vert \vert z \vert +\sum_{k=2}^{\infty} \vert a_{k} \vert \vert z \vert ^{k}\leq1 \quad\textit{for } \vert z \vert =r \leq\frac{\sqrt{17}-3}{4}. $$

*The radius* $r=\frac{\sqrt{17}-3}{4}$ *is the best possible*.

### Proof

By assumption, $f(z)=\sum_{k=0}^{\infty}a_{k}z^{k}$ is analytic in *D* and $|f(z)|<1$ in *D*. Since $f(0)=a_{0}$, by the Schwarz–Pick lemma, we obtain, for $z\in D$,

$$ \frac{ \vert f(z)-a_{0} \vert }{ \vert 1-\overline{a_{0}}f(z) \vert }\leq \vert z \vert , \qquad \bigl\vert f'(z) \bigr\vert \leq\frac {1- \vert f(z) \vert ^{2}}{1- \vert z \vert ^{2}}. $$

Thus it follows from the above inequality and Lemma 1.2 that, for $z=re^{i\theta}\in D$,

$$ \bigl\vert f(z) \bigr\vert \leq\frac{r+ \vert a_{0} \vert }{1+r \vert a_{0} \vert },\qquad \vert a_{k} \vert \leq1- \vert a_{0} \vert ^{2} $$

for $k=1,2,\ldots $ .

Using these inequalities, we have

2.1 $$\begin{aligned} & \bigl\vert f(z) \bigr\vert + \bigl\vert f'(z) \bigr\vert r+\sum_{k=2}^{\infty} \vert a_{k} \vert r^{k} \\ &\quad\leq\frac{r}{1-r^{2}}\bigl(1- \bigl\vert f(z) \bigr\vert ^{2} \bigr)+ \bigl\vert f(z) \bigr\vert +\bigl(1- \vert a_{0} \vert ^{2}\bigr)\frac {r^{2}}{1-r} \\ &\quad\leq\frac{r}{1-r^{2}}\biggl[1-\biggl(\frac{r+ \vert a_{0} \vert }{1+ \vert a_{0} \vert r}\biggr)^{2} \biggr]+\frac {r+ \vert a_{0} \vert }{1+ \vert a_{0} \vert r}+\bigl(1- \vert a_{0} \vert ^{2}\bigr)\frac{r^{2}}{1-r} \\ &\quad=\frac{ \vert a_{0} \vert +2r+ \vert a_{0} \vert r^{2}}{(1+ \vert a_{0} \vert r)^{2}}+\bigl(1- \vert a_{0} \vert ^{2} \bigr)\frac {r^{2}}{1-r}, \end{aligned}$$

where the second inequality holds for any $r\in[0,\sqrt{2}-1)$, since $\frac{1-r^{2}}{2r}\geq1$ if $r\in[0,\sqrt{2}-1]$.

Notice $|a_{0}|<1$, we know (2.1) is smaller than or equal to 1 provided $\varphi(r)\leq0$, where

$$\begin{aligned} \varphi (r)&=\bigl( \vert a_{0} \vert +2r+ \vert a_{0} \vert r^{2}\bigr) (1-r)+\bigl(1+ \vert a_{0} \vert r\bigr)^{2}\bigl(1- \vert a_{0} \vert ^{2}\bigr)r^{2}-\bigl(1+ \vert a_{0} \vert r\bigr)^{2}(1-r) \\ &=\bigl(1- \vert a_{0} \vert \bigr)\bigl[-1+3r+\bigl(2 \vert a_{0} \vert -1\bigr)r^{2}+ \vert a_{0} \vert \bigl(2 \vert a_{0} \vert +1\bigr)r^{3}+ \vert a_{0} \vert ^{2}\bigl(1+ \vert a_{0} \vert \bigr)r^{4}\bigr] \\ &\leq\bigl(1- \vert a_{0} \vert \bigr) \bigl(-1+3r+r^{2}+3r^{3}+2r^{4} \bigr) \\ &=\bigl(1- \vert a_{0} \vert \bigr)2\bigl(1+r^{2} \bigr) \biggl(r+\frac{\sqrt{17}+3}{4}\biggr) \biggl(r-\frac{\sqrt{17}-3}{4}\biggr). \end{aligned}$$

Now, $\varphi(r)\leq0$ if $\eta(r):=(1+r^{2})(r+\frac{\sqrt {17}+3}{4})(r-\frac{\sqrt{17}-3}{4})\leq0$, which holds for $r\leq\frac {\sqrt{17}-3}{4}$. The first part of the theorem follows.

To show the sharpness of the number $r=\frac{\sqrt{17}-3}{4}$, we let $a\in[0,1)$ and consider the function

$$\begin{aligned} f(z)=\frac{a-z}{1-az}=a-\bigl(1-a^{2}\bigr)\sum _{k=1}^{\infty}a^{k-1}z^{k},\quad z\in D. \end{aligned}$$

For this function, we find that

2.2 $$\begin{aligned} \bigl\vert f(-r) \bigr\vert + \bigl\vert f'(-r) \bigr\vert r+\sum_{k=2}^{\infty} \vert a_{k} \vert r^{k}=\frac{a+r}{1+ar}+\frac {1-a^{2}}{(1+ar)^{2}}r+ \bigl(1-a^{2}\bigr)\frac{ar^{2}}{1-ar}. \end{aligned}$$

The last expression is larger than 1 if and only if

2.3 $$\begin{aligned} (1-a) \bigl(-1+(2+a)r+a^{2}r^{2}+a^{2}(2a+1)r^{3}+a^{3}(1+a)r^{4} \bigr)> 0. \end{aligned}$$

Let $P_{3}(a,r)=-1+(2+a)r+a^{2}r^{2}+a^{2}(2a+1)r^{3}+a^{3}(1+a)r^{4}$. After elementary calculation, we find that $\frac{\partial P_{3}}{\partial a}=r+2ar^{2}+6a^{2}r^{3}+2ar^{3}+3a^{2}r^{4}+4a^{3}r^{4}$ is equal to or greater than 0 for any $r\in[0,1)$. The latter equation implies that

$$P_{3}(a,r)\leq P_{3}(1,r)=-1+3r+r^{2}+3r^{3}+2r^{4}=2 \bigl(1+r^{2}\bigr) \biggl(r+\frac {\sqrt{17}+3}{4}\biggr) \biggl(r- \frac{\sqrt{17}-3}{4}\biggr). $$

Therefore, Eq. (2.2) is smaller than or equal to 1 for all $a\in[0,1)$, only in the case when $r\leq\frac{\sqrt{17}-3}{4}$. Finally, it also suggests that $a\rightarrow1$ in (2.3) shows that Eq. (2.2) is larger than 1 if $r>\frac{\sqrt {17}-3}{4}$. This proves the sharpness. □

Next, we discuss the Bohr-type radius when the coefficients of the series of missing series are completely replaced by the higher order derivatives.

### Theorem 2.2

*Suppose that* $N (\geq2)$ *is an integer*, $f(z)=\sum_{k=0}^{\infty}a_{k}z^{k}$ *is analytic in* *D* *and* $|f(z)|<1$ *in* *D*. *Then*

$$ \bigl\vert f(z) \bigr\vert +\sum_{k=N}^{\infty} \biggl\vert \frac{f^{(k)}(z)}{k!} \biggr\vert \vert z \vert ^{k} \leq 1 \quad\textit{for } \vert z \vert =r\leq R_{N}, $$

*where* $R_{N}$ *is the minimum positive root of the equation* $\psi _{N}(r)=(1+r)(1-2r)(1-r)^{N-1}-2r^{N}=0$. *The radius* $R_{N}$ *is the best possible*.

### Proof

By simple calculations we can know that

$$ r\leq R_{N}< 1/2 \quad\mbox{if and only if}\quad \frac {2r^{N}}{(1+r)(1-2r)(1-r)^{N-1}}\leq1. $$

By assumption, $f(z)=\sum_{k=0}^{\infty}a_{k}z^{k}$ is analytic in *D* and $|f(z)|<1$ in *D*. Since $f(0)=a_{0}$, it follows from the Schwarz–Pick lemma and Lemma 1.4 that, for $z=re^{i\theta}\in D$,

$$ \bigl\vert f(z) \bigr\vert \leq\frac{r+ \vert a_{0} \vert }{1+ \vert a_{0} \vert r} \quad\mbox{and}\quad \bigl\vert f^{(k)}(z) \bigr\vert \leq \frac{k!(1- \vert f(z) \vert ^{2})}{(1- \vert z \vert ^{2})^{k}}\bigl(1+ \vert z \vert \bigr)^{k-1}\quad\textit {for } k=1,2,\ldots. $$

Using these inequalities, we have

2.4 $$\begin{aligned} & \bigl\vert f(z) \bigr\vert +\sum_{k=N}^{\infty} \biggl\vert \frac{f^{(k)}(z)}{k!} \biggr\vert r^{k} \\ &\quad\leq \bigl\vert f(z) \bigr\vert +\sum_{k=N}^{\infty} \frac {(1- \vert f(z) \vert ^{2})}{(1- \vert z \vert ^{2})^{k}}\bigl(1+ \vert z \vert \bigr)^{k-1}r^{k} \\ &\quad = \bigl\vert f(z) \bigr\vert +\bigl(1- \bigl\vert f(z) \bigr\vert ^{2}\bigr)\sum_{k=N}^{\infty} \frac {(1+r)^{k-1}r^{k}}{(1-r^{2})^{k}} \\ &\quad \leq \bigl\vert f(z) \bigr\vert +\bigl(1- \bigl\vert f(z) \bigr\vert ^{2}\bigr)\frac {r^{N}}{(1+r)(1-2r)(1-r)^{N-1}} \\ &\quad= \bigl\vert f(z) \bigr\vert -\frac{r^{N}}{(1+r)(1-2r)(1-r)^{N-1}} \bigl\vert f(z) \bigr\vert ^{2}+\frac {r^{N}}{(1+r)(1-2r)(1-r)^{N-1}} \\ &\quad\leq \frac {( \vert a_{0} \vert +r)(1+ \vert a_{0} \vert r)(1-r)^{N}(1-2r)+(1- \vert a_{0} \vert ^{2})(1-r)^{2}r^{N}}{(1-r)^{N}(1-2r)(1+ \vert a_{0} \vert r)^{2}}:=\omega _{N}(r) \end{aligned}$$

for $0\leq r\leq R_{N}<1/2$.

Now, $\omega_{N}(r)\leq1$ if $\nu_{N}(r)\leq0$, where

$$\begin{aligned} \nu_{N}(r) ={}& \bigl( \vert a_{0} \vert +r \bigr) \bigl(1+ \vert a_{0} \vert r\bigr) (1-r)^{N}(1-2r)+ \bigl(1- \vert a_{0} \vert ^{2}\bigr) (1-r)^{2}r^{N} \\ &{}-(1-r)^{N}(1-2r) \bigl(1+ \vert a_{0} \vert r \bigr)^{2} \\ ={}&\bigl(1- \vert a_{0} \vert \bigr)\bigl[\bigl(-1+\bigl(3- \vert a_{0} \vert \bigr)r+\bigl(3 \vert a_{0} \vert -2\bigr)r^{2}-2 \vert a_{0} \vert r^{3} \bigr) (1-r)^{N}\\ &{}+\bigl(1+ \vert a_{0} \vert \bigr)r^{N}(1-r)^{2}\bigr] \\ ={}&\bigl(1- \vert a_{0} \vert \bigr)\bigl[\bigl(-1+3r-2r^{2} \bigr) (1-r)^{N}+r^{N}(1-r)^{2}\bigr] \\ &{}+\bigl(1- \vert a_{0} \vert \bigr) \vert a_{0} \vert r(1-r)^{2}\bigl[r^{N-1}-(1-2r) (1-r)^{N-1} \bigr]. \end{aligned}$$

Now we split all this into two cases to prove that $\nu_{N}(r)\leq0$ for $r\leq R_{N}$.

Case 1. $r\leq R_{N,1}$, where $R_{N,1}$ is the minimum positive root of the equation $\varphi_{N}(r)=(1-2r)(1-r)^{N-1}-r^{N-1}=0$. Since $r^{N-1}-(1-2r)(1-r)^{N-1}\leq0$ and $|a_{0}|<1$, we have

$$\begin{aligned} \nu_{N}(r) &\leq \bigl(1- \vert a_{0} \vert \bigr) (1-r)^{2}\bigl[r\cdot r^{N-1}-(1-2r) (1-r)^{N-1}\bigr] \\ &\leq \bigl(1- \vert a_{0} \vert \bigr) (1-r)^{2}\bigl[r^{N-1}-(1-2r) (1-r)^{N-1}\bigr]\leq0. \end{aligned}$$

Case 2. $R_{N,1}< r\leq R_{N}$. Notice that $R_{N,1}< R_{N}$ and $r^{N-1}-(1-2r)(1-r)^{N-1}>0$ for $r>R_{N,1}$, we have

$$\begin{aligned} \nu_{N}(r) \leq{}&\bigl(1- \vert a_{0} \vert \bigr) \bigl[\bigl(-1+3r-2r^{2}\bigr) (1-r)^{N}+r^{N}(1-r)^{2} \bigr] \\ &{}+\bigl(1- \vert a_{0} \vert \bigr)r(1-r)^{2} \bigl[r^{N-1}-(1-2r) (1-r)^{N-1}\bigr] \\ \leq{}&\bigl(1- \vert a_{0} \vert \bigr) (1-r)^{2} \bigl[2r^{N}-(1+r) (1-2r) (1-r)^{N-1}\bigr]\leq0. \end{aligned}$$

The first part of the theorem follows.

To show the sharpness of the number $R_{N}$, we let $a\in[0,1)$ and consider the function

$$\begin{aligned} f(z)=\frac{a-z}{1-az}=a-\bigl(1-a^{2}\bigr)\sum _{k=1}^{\infty}a^{k-1}z^{k},\quad z\in D. \end{aligned}$$

For this function, we find that

2.5 $$\begin{aligned} &\bigl\vert f(r) \bigr\vert +\sum_{k=N}^{\infty} \biggl\vert \frac{f^{(k)}(r)}{k!} \biggr\vert r^{k} \\ &\quad = \bigl\vert f(r) \bigr\vert +\sum_{k=N}^{\infty} \frac {a^{k-1}(1-a^{2})}{(1-ar)^{k+1}}r^{k} \\ &\quad =\frac{a-r}{1-ar}+\bigl(1-a^{2}\bigr)\frac {a^{N-1}r^{N}}{(1-ar)^{N}(1-2ar)} \quad\biggl(\mbox{when } r< \frac{1}{2a}\biggr). \end{aligned}$$

The last expression is larger than 1 if and only if

2.6 $$\begin{aligned} (1-a)\bigl[\bigl(-1+(2a-1)r+2ar^{2}\bigr) (1-ar)^{N-1}+(1+a)a^{N-1}r^{N} \bigr]> 0. \end{aligned}$$

Let $P_{4}(a,r)=(-1+(2a-1)r+2ar^{2})(1-ar)^{N-1}+(1+a)a^{N-1}r^{N}$. After elementary calculation, we find that $\frac{\partial P_{4}}{\partial a}=(2r+2r^{2})(1-ar)^{N-1}+r(N-1)(1+r)(1-2ar)(1-ar)^{N-2}+a^{N-1}r^{N}+(1+a)(N-1)a^{N-2}r^{N}$ is equal to or greater than 0 for any $r<\frac{1}{2}$. The latter equation implies that

$$\begin{aligned} P_{4}(a,r)\leq P_{4}(1,r)=\bigl(-1+r+2r^{2} \bigr) (1-r)^{N-1}+2r^{N}=2r^{N}-(r+1) (1-2r) (1-r)^{N-1} \end{aligned}$$

holds for $r<\frac{1}{2}$. Therefore, Eq. (2.5) is smaller than or equal to 1 for all $a\in[0,1)$, only in the case when $r\leq R_{N}$.

Finally, allowing $a\rightarrow1$ in (2.6) shows that Eq. (2.5) is larger than 1 if $r>R_{N}$. This proves the sharpness. □

### Corollary 2.3

*Suppose that* $f(z)=\sum_{k=0}^{\infty}a_{k}z^{k}$ *is analytic in* *D* *and* $|f(z)|<1$ *in* *D*. *Then*

$$ \bigl\vert f(z) \bigr\vert ^{2}+\sum _{k=N}^{\infty} \biggl\vert \frac{f^{(k)}(z)}{k!} \biggr\vert \vert z \vert ^{k}\leq1 \quad\textit{for } \vert z \vert =r\leq R'_{N}, $$

*where* $R'_{N}$ *is the positive root of the equation* $(1+r)(1-2r)(1-r)^{N-1}-r^{N}=0$. *The radius* $R'_{N}$ *is the best possible*.

### Proof

By simple calculations we can know that

$$ r\leq R'_{N} \quad\mbox{if and only if}\quad \frac {(1+r)(1-r)^{N}(1-2r)-r^{N}(1-r)}{(1+r)(1-2r)(1-r)^{N}} \geq0. $$

In analogy to the calculation of Theorem 2.2, we have

2.7 $$\begin{aligned} & \bigl\vert f(z) \bigr\vert ^{2}+\sum _{k=N}^{\infty} \biggl\vert \frac{f^{(k)}(z)}{k!} \biggr\vert r^{k} \\ &\quad \leq \bigl\vert f(z) \bigr\vert ^{2}+\sum _{k=N}^{\infty}\frac {(1- \vert f(z) \vert ^{2})}{(1- \vert z \vert ^{2})^{k}}\bigl(1+ \vert z \vert \bigr)^{k-1}r^{k} \\ &\quad \leq\biggl(1-\frac{r^{N}(1-r)}{(1+r)(1-2r)(1-r)^{N}}\biggr) \bigl\vert f(z) \bigr\vert ^{2}+\frac {r^{N}(1-r)}{(1+r)(1-2r)(1-r)^{N}} \\ &\quad \leq\frac {( \vert a_{0} \vert +r)^{2}(1-r)^{N}(1-2r)+(1- \vert a_{0} \vert ^{2})r^{N}(1-r)^{2}}{(1-r)^{N}(1+ \vert a_{0} \vert r)^{2}(1-2r)}. \end{aligned}$$

So (2.7) is smaller than or equal to 1 provided $\omega _{N}(r)\leq1$, where

$$\begin{aligned} \omega_{N}(r):=\frac {( \vert a_{0} \vert +r)^{2}(1-r)^{N}(1-2r)+(1- \vert a_{0} \vert ^{2})r^{N}(1-r)^{2}}{(1-r)^{N}(1+ \vert a_{0} \vert r)^{2}(1-2r)}. \end{aligned}$$

Now, $\omega_{N}(r)\leq1$ if $\nu_{N}(r)\leq0$, where

$$\begin{aligned} \nu_{N}(r) &=\bigl( \vert a_{0} \vert +r \bigr)^{2}(1-r)^{N}(1-2r)+\bigl(1- \vert a_{0} \vert ^{2}\bigr)r^{N}(1-r)^{2}-(1-r)^{N} \bigl(1+ \vert a_{0} \vert r\bigr)^{2}(1-2r) \\ &=\bigl(1- \vert a_{0} \vert ^{2}\bigr) \bigl[(1-r)^{N}\bigl(-1+r^{2}+2r-2r^{3} \bigr)+r^{N}(1-r)^{2}\bigr] \\ &=\bigl(1- \vert a_{0} \vert ^{2}\bigr) \bigl[(1-r)^{N}(1-r) (1+r) (2r-1)+r^{N}(1-r)^{2} \bigr]. \end{aligned}$$

Now, $\nu_{N}(r)\leq0$ if $(1+r)(1-2r)(1-r)^{N-1}-r^{N}\geq0$, which holds for $r\leq R'_{N}$, where $R'_{N}$ is as in the statement of the theorem.

To show the sharpness of the number $R'_{N}$, we let $a\in[0,1)$ and consider the function

$$\begin{aligned} f(z)=\frac{a-z}{1-az}=a-\bigl(1-a^{2}\bigr)\sum _{k=1}^{\infty}a^{k-1}z^{k},\quad z\in D. \end{aligned}$$

For this function, we find that

2.8 $$\begin{aligned} \bigl\vert f(r) \bigr\vert ^{2}+\sum _{k=N}^{\infty} \biggl\vert \frac{f^{(k)}(r)}{k!} \biggr\vert r^{k}&= \bigl\vert f(r) \bigr\vert ^{2}+\sum _{k=N}^{\infty}\frac {a^{k-1}(1-a^{2})}{(1-ar)^{k+1}}r^{k} \\ &=\frac{(a-r)^{2}(1-ar)^{N-2}(1-2ar)+(1-a^{2})a^{N-1}r^{N}}{(1-ar)^{N}(1-2ar)}, \end{aligned}$$

(2.8) is larger than 1 if and only if

2.9 $$\begin{aligned} \bigl(1-a^{2}\bigr)\bigl[\bigl(-1+2ar+r^{2}-2ar^{3} \bigr) (1-ar)^{N-2}+a^{N-1}r^{N}\bigr]> 0. \end{aligned}$$

In analogy to the processing methods of Theorem 2.2. After elementary calculation, we find that allowing $a\rightarrow1$ in (2.9), it follows that Eq. (2.8) is larger than 1 if $r>R'_{N}$. This proves the sharpness and we complete the proof of Corollary 2.3. □

Applying a method similar to Theorem 2.2, we may obtain the following corollary.

### Corollary 2.4

*Suppose that* $f(z)=\sum_{k=0}^{\infty}a_{k}z^{k}$ *is analytic in* *D* *and* $|f(z)|<1$ *in* *D*. *Then*

$$ \sum_{k=0}^{\infty} \biggl\vert \frac{f^{(k)}(z)}{k!} \biggr\vert \vert z \vert ^{k}\leq1 \quad\textit {for } \vert z \vert =r\leq\frac{\sqrt{17}-3}{4}. $$

*The radius* $r=\frac{\sqrt{17}-3}{4}$ *is the best possible*.

In analogy to Theorem C, we now consider the Bohr-type radius when conditions of $|f(z)|<1$ are replaced by $\operatorname{ Re} f(z)\leq1$ and $f(0)=a_{0}$ is positive.

### Theorem 2.5

*If* $f(z)=\sum_{k=0}^{\infty}a_{k}z^{k}$ *is analytic in* *D* *satisfying* $\operatorname{ Re} f(z)\leq1$ *in* *D* *and* $f(0)=a_{0}$ *is positive*, *then*

2.10 $$ \bigl\vert f(z) \bigr\vert +\sum_{k=1}^{\infty} \vert a_{nk} \vert \vert z \vert ^{nk}\leq1 \quad\textit{for } \vert z \vert =r\leq R_{n}, $$

*where* $R_{n}$ *is the positive root of the equation* $\varphi_{n}(r)=0$, $\varphi_{n}(r)=r^{n+1}+3r^{n}+r-1$. *The radius* $R_{n}$ *is the best possible*.

### Proof

By assumption, $f(z)=\sum_{k=0}^{\infty}a_{nk}z^{nk}$ is analytic and $\operatorname{ Re} f(z)\leq1$ in *D*.

Since $f(0)=a_{0}$ is positive. Applying the result of Lemma 1.5 to $p(z)=1-f(z)$ and the Schwarz–Pick lemma that, for $z=re^{i\theta}\in D$, we have

$$ \vert a_{nk} \vert \leq2(1-a_{0})\quad \mbox{for } k=1,2, \ldots $$

and

$$ \bigl\vert f(z) \bigr\vert \leq\frac{r+a_{0}}{1+ra_{0}}. $$

Using the last two inequalities, we have

2.11 $$\begin{aligned} \bigl\vert f(z) \bigr\vert +\sum_{k=1}^{\infty} \vert a_{nk} \vert r^{nk}\leq\frac {r+a_{0}}{1+a_{0}r}+2(1-a_{0}) \frac{r^{n}}{1-r^{n}}, \end{aligned}$$

for which (2.11) is smaller than or equal to 1 provided $\phi (r)\leq0$, where

$$\begin{aligned} \phi (r)&=(r+a_{0}) \bigl(1-r^{n} \bigr)+2(1-a_{0})r^{n}(1+a_{0}r)-(1+a_{0}r) \bigl(1-r^{n}\bigr) \\ &=(1-a_{0})\bigl[(2a_{0}-1)r^{n+1}+3r^{n}-1+r \bigr] \\ &\leq (1-a_{0}) \bigl(r^{n+1}+3r^{n}+r-1\bigr)\quad \mbox{since } \vert a_{0} \vert < 1. \end{aligned}$$

Now, $\phi(r)\leq0$ if $\psi(r):=r^{n+1}+3r^{n}+r-1\leq0$, which holds for $r\leq R_{n}$. This completes the proof of inequality (2.10).

To shows that the radius $r=R_{n}$ is the best possible, we let $a\in [0,1)$ and consider the function

$$\begin{aligned} f(z)=\frac{a-z}{1-az}=a-\bigl(1-a^{2}\bigr)\sum _{k=1}^{\infty}a^{k-1}z^{k}, \quad z\in D. \end{aligned}$$

For this function, we find that

2.12 $$\begin{aligned} \bigl\vert f(-r) \bigr\vert +\sum_{k=1}^{\infty} \vert a_{nk} \vert r^{nk}=\frac {a+r}{1+ar}+ \bigl(1-a^{2}\bigr)\frac{a^{n-1}r^{n}}{1-a^{n}r^{n}}, \quad\mbox{where } r= \vert z \vert . \end{aligned}$$

We claim that, for every *r* such that $R_{n}< r<1$, there is a such that $0< a<1$, and

2.13 $$\begin{aligned} \frac{(a+r)(1-a^{n}r^{n})+(1-a^{2})(1+ar)a^{n-1}r^{n}}{(1+ar)(1-a^{n}r^{n})}>1. \end{aligned}$$

Indeed, inequality (2.13) is equivalent to the inequality

2.14 $$\begin{aligned} (1-a)\bigl[\bigl(a^{n-1}+2a^{n}\bigr)r^{n}+a^{n+1}r^{n+1}+r-1 \bigr]> 0. \end{aligned}$$

Let $P_{1}(a,r)=(a^{n-1}+2a^{n})r^{n}+a^{n+1}r^{n+1}+r-1$ denote a part of the left-hand side of (2.14). After elementary calculation, we find that $\frac{\partial P_{1}}{\partial a}\geq0$ apparently. The latter inequality implies that $P_{1}(a,r)\leq P_{1}(1,r)=r^{n+1}+3r^{n}+r-1$ holds for all $r\in[0,1)$. Therefore, Eq. (2.12) is smaller than or equal to 1 for all $a\in[0,1)$, only in the case when $r\leq R_{n}$.

Finally, allowing $a\rightarrow1$ in (2.14) shows that Eq. (2.13) is larger than 1 if $r>R_{n}$. This proves the sharpness. □

Setting $n=1$ in Theorem 2.5, we have the following corollary.

### Corollary 2.6

*If* $f(z)=\sum_{k=0}^{\infty}a_{k}z^{k}$ *is analytic in* *D* *satisfying* $\operatorname{ Re} f(z)\leq1$ *in* *D* *and* $f(0)=a_{0}$ *is positive*, *then*

$$ \bigl\vert f(z) \bigr\vert +\sum_{k=1}^{\infty} \vert a_{k} \vert \vert z \vert ^{k}\leq1\quad \textit{for } \vert z \vert =r\leq \sqrt{5}-2, $$

*where the radius* $\sqrt{5}-2$ *is the best possible*.

### Remark 2.7

By simple calculation, we can know the Bohr-type radius in Theorem 2.5 with the condition of $\operatorname{ Re} f(z)\leq1$ and $f(0)=a_{0}>0$ is the same as the condition of $|f(z)|<1$.

Finally, we consider a new Bohr-type radius of the alternating series associated the Taylor series of analytic functions where $|a_{0}|$ is replaced by $|f(z)|$. We have

$$ R_{f}(z)= \bigl\vert f(z) \bigr\vert +\sum _{k=1}^{\infty}(-1)^{k} \vert a_{k} \vert \vert z \vert ^{k}. $$

### Lemma 2.8

*Suppose that* $f(z)=\sum_{k=0}^{\infty}a_{k}z^{k}$ *is analytic in the unit disk D* *and* $|f(z)|<1$ *in D*. *Then*

2.15 $$ \bigl\vert f(z) \bigr\vert +\sum_{k=1}^{\infty} \vert a_{2k} \vert \vert z \vert ^{2k}\leq1 \quad\textit{for } \vert z \vert =r\leq\sqrt{2}-1. $$

*The radius* $r=\sqrt{2}-1$ *is the best possible*.

### Proof

By assumption, $f(z)=\sum_{k=0}^{\infty}a_{k}z^{k}$ is analytic and $|f(z)|<1$ in *D*. Since $f(0)=a_{0}$, it follows from Lemma 1.2 and the Schwarz–Pick lemma that, for $z=re^{i\theta}\in D$,

$$ \vert a_{k} \vert \leq1- \vert a_{0} \vert ^{2} \quad\mbox{for } k=1,2,\ldots $$

and

$$ \bigl\vert f(z) \bigr\vert \leq\frac{r+ \vert a_{0} \vert }{1+r \vert a_{0} \vert }. $$

Using the last two inequalities, we have

2.16 $$\begin{aligned} \bigl\vert f(z) \bigr\vert +\sum_{k=1}^{\infty} \vert a_{2k} \vert r^{2k}\leq \frac {r+ \vert a_{0} \vert }{1+ \vert a_{0} \vert r}+ \bigl(1- \vert a_{0} \vert ^{2}\bigr)\frac{r^{2}}{1-r^{2}}, \end{aligned}$$

and (2.16) is smaller than or equal to 1 provided $\phi(r)\leq 0$, where

$$\begin{aligned} \phi (r)&=\bigl(r+ \vert a_{0} \vert \bigr) \bigl(1-r^{2}\bigr)+\bigl(1- \vert a_{0} \vert ^{2}\bigr)r^{2}\bigl(1+ \vert a_{0} \vert r \bigr)-\bigl(1+ \vert a_{0} \vert r\bigr) \bigl(1-r^{2} \bigr) \\ &=\bigl(1- \vert a_{0} \vert \bigr)\bigl[-1+r+\bigl(2+ \vert a_{0} \vert \bigr)r^{2}+\bigl( \vert a_{0} \vert ^{2}+ \vert a_{0} \vert -1\bigr)r^{3} \bigr] \\ &\leq\bigl(1- \vert a_{0} \vert \bigr) \bigl(3r^{2}+r-1+r^{3} \bigr) \\ &=\bigl(1- \vert a_{0} \vert \bigr) (r+1+\sqrt{2}) (r+1- \sqrt{2}) (r+1), \quad\mbox{since } \vert a_{0} \vert < 1. \end{aligned}$$

Now, $\phi(r)\leq0$ if $\psi(r):=(r+1+\sqrt{2})(r+1-\sqrt{2})(r+1)\leq 0$, which holds for $r\leq\sqrt{2}-1$. This completes the proof of inequality (2.15).

To shows that the radius $r=\sqrt{2}-1$ is the best possible, we let $a\in[0,1)$ and consider the function

$$\begin{aligned} f(z)=\frac{a-z}{1-az}=a-\bigl(1-a^{2}\bigr)\sum _{k=1}^{\infty}a^{k-1}z^{k},\quad z\in D. \end{aligned}$$

For this function, we find that

2.17 $$\begin{aligned} \bigl\vert f(-r) \bigr\vert +\sum_{k=1}^{\infty} \vert a_{2k} \vert r^{2k}=\frac {a+r}{1+ar}+ \bigl(1-a^{2}\bigr)\frac{ar^{2}}{1-a^{2}r^{2}},\quad \mbox{where } r= \vert z \vert . \end{aligned}$$

We claim that, for every *r* such that $\sqrt{2}-1< r<1$, there is a such that $0< a<1$, and

2.18 $$\begin{aligned} \frac{(a+r)(1-ar)+(1-a^{2})ar^{2}}{(1+ar)(1-ar)}>1. \end{aligned}$$

Indeed, inequality (2.18) is equivalent to the inequality

2.19 $$\begin{aligned} (1-a)\bigl[-1+(1+a)r+a^{2}r^{2}\bigr]> 0. \end{aligned}$$

Let $P_{1}(a,r)=-1+(1+a)r+a^{2}r^{2}$ denote a part of the left-hand side of (2.19). After elementary calculation, we find that $\frac {\partial P_{1}}{\partial a}=r+2ar^{2}\geq0$. The latter inequality implies that $P_{1}(a,r)\leq P_{1}(1,r)=-1+2r+r^{2}$ holds for all $r\in [0,1)$. Therefore, Eq. (2.17) is smaller than or equal to 1 for all $a\in[0,1)$, only in the case when $r\leq\sqrt{2}-1$.

Finally, allowing $a\rightarrow1$ in (2.19) shows that Eq. (2.18) is larger than 1 if $r>\sqrt{2}-1$. This proves the sharpness. □

### Theorem 2.9

*Suppose that* $f(z)=\sum_{k=0}^{\infty}a_{k}z^{k}$ *is analytic in* *D* *and* $|f(z)|<1$ *in* *D*. *Then*

2.20 $$ \Biggl| \bigl\vert f(z) \bigr\vert +\sum_{k=1}^{\infty}(-1)^{k} \vert a_{k} \vert \vert z \vert ^{k}\Biggr|\leq1\quad \textit{for } \vert z \vert =r \leq\sqrt{2}-1. $$

### Proof

By the proof of Lemma 2.8, we have

2.21 $$\begin{aligned} \bigl\vert f(z) \bigr\vert +\sum_{k=1}^{\infty}(-1)^{k} \vert a_{k} \vert r^{k} &\leq\frac {r+ \vert a_{0} \vert }{1+ \vert a_{0} \vert r}+\sum _{k=1}^{\infty} \vert a_{2k} \vert r^{2k}-\sum_{k=1}^{\infty} \vert a_{2k-1} \vert r^{2k-1} \\ &\leq\frac{r+ \vert a_{0} \vert }{1+ \vert a_{0} \vert r}+\sum_{k=1}^{\infty } \vert a_{2k} \vert r^{2k} \\ &\leq\frac{r+ \vert a_{0} \vert }{1+ \vert a_{0} \vert r}+\bigl(1- \vert a_{0} \vert ^{2}\bigr)\frac{r^{2}}{1-r^{2}}. \end{aligned}$$

We know that Eq. (2.21) is smaller than or equal to 1, which holds for $r\leq\sqrt{2}-1$ and for all $a\in[0,1)$.

To find a lower bound for $R_{f}(z)$, we consider the following chain of relations:

$$\begin{aligned} R_{f}(z)&= \bigl\vert f(z) \bigr\vert +\sum _{k=1}^{\infty} \vert a_{2k} \vert r^{2k}-\sum_{k=1}^{\infty } \vert a_{2k-1} \vert r^{2k-1} \\ &\geq -\sum_{k=1}^{\infty} \vert a_{2k-1} \vert r^{2k-1} = -\Biggl( \vert a_{1} \vert r+\sum_{k=1}^{\infty} \vert a_{2k+1} \vert r^{2k+1}\Biggr) \\ &\geq - \Biggl(\bigl(1- \vert a_{0} \vert ^{2}\bigr)r+\sum_{k=1}^{\infty} \vert a_{2k+1} \vert r^{2k} \Biggr) \\ &\geq-\Biggl(\frac{r+ \vert a_{0} \vert }{1+r \vert a_{0} \vert }+\sum_{k=1}^{\infty} \vert a_{2k+1} \vert r^{2k}\Biggr), \end{aligned}$$

where the last inequality is obtained by a simple calculation.

Combining this with (2.21), we conclude that $R_{f}(z)\geq-1$ for all $r\leq\sqrt{2}-1$. This completes the proof of inequality (2.20). □

Notice that we have not proved that the number $r=\sqrt{2}-1$ is the best possible in Theorem 2.9, therefore the following problem remains open.

### Problem 2.10

Find the largest radius $r_{0}$ for the class of analytic functions $f(z)=\sum_{k=0}^{\infty}a_{k}z^{k}$ in *D* with $|f(z)|<1$ in *D* such that

$$ \Biggl| \bigl|f(z) \bigr| +\sum_{k=1}^{\infty}(-1)^{k}|a_{k}||z|^{k}\biggr| \leq1 \quad\mbox{for } \vert z \vert =r\leq r_{0}. $$

## Conclusion

From the results that we have given in this paper, we can get the exact Bohr-type radius when we replace the coefficient of Bohr’s inequality with $f(z)$ or its higher order derivatives, and we conclude that the Bohr-type radius obtained after the change of coefficients is smaller than the Bohr radius.
